# Identification of Indazole-Based Thiadiazole-Bearing Thiazolidinone Hybrid Derivatives: Theoretical and Computational Approaches to Develop Promising Anti-Alzheimer’s Candidates

**DOI:** 10.3390/ph16121667

**Published:** 2023-11-30

**Authors:** Yousaf Khan, Shoaib Khan, Rafaqat Hussain, Wajid Rehman, Aneela Maalik, Urooba Gulshan, Mohamed W. Attwa, Hany W. Darwish, Hazem A. Ghabbour, Nawab Ali

**Affiliations:** 1 Department of Chemistry, COMSATS University Islamabad, Islamabad 45550, Pakistan; yousaf7n@gmail.com (Y.K.); uroobagulshan@gmail.com (U.G.); 2 Department of Chemistry, Abbottabad University of Science and Technology (AUST), Abbottabad 22500, Pakistan; shoaibkhanswati@gmail.com; 3 Department of Chemistry, Hazara University, Mansehra 21120, Pakistan; sono_waj@yahoo.com; 4 Department of Pharmaceutical Chemistry, College of Pharmacy, King Saud University, Riyadh 11451, Saudi Arabia; mzeidan@ksu.edu.sa (M.W.A.); hdarwish@ksu.edu.sa (H.W.D.); 5 School of Health and Biomedical Sciences, RMIT University, Melbourne 3083, Australia; hazem.ghabbour@rmit.edu.au; 6 Shangai Key Laboratory of Functional Material Chemistry, School of Chemistry and Molecular Engineering, East China University of Science and Technology, Meilong Road 130, Shanghai 200237, China; nawabsci@yahoo.com

**Keywords:** indazole, thiadiazole, thiazolidinone, SAR, acetylcholinesterase, butyrylcholinesterase, molecular docking

## Abstract

A hybrid library of compounds based on indazole-based thiadiazole containing thiazolidinone moieties (**1**–**17**) was synthesized. The synthesized compounds were screened in vitro for their inhibition profile against targetedacetylcholinesterase (AChE) and butyrylcholinesterase (BuChE) activities. All the derivatives demonstrated a varied range of inhibitory activities having IC_50_ values ranging from 0.86 ± 0.33 μM to 26.73 ± 0.84 μM (AChE) and 0.89 ± 0.12 μM to 27.08 ± 0.19 μM (BuChE), respectively. The results obtained were compared with standard Donepezil drugs (IC_50_ = 1.26 ± 0.18 μM for AChE) and (1.35 ± 0.37 μM for BuChE), respectively. Specifically, the derivatives **1**–**17**, **1**, **9**, and **14** were found to be significantly active, with IC_50_ values of 0.86 ± 0.30, 0.92 ± 0.10, and 1.10 ± 0.37 μM (against AChE) and 0.89 ± 0.12, 0.98 ± 0.48 and 1.19 ± 0.42 μM (against BuChE), respectively.The structure–activity relationship (SAR) studies revealed that derivatives bearing *para*-CF_3_, *ortho*-OH, and *para*-F substitutions on the phenyl ring attached to the thiadiazole skeleton, as well as *meta*-Cl, -NO_2_, and *para*-chloro substitutions on the phenyl ring, having a significant effect on inhibitory potential. The synthesized scaffolds have been further characterized by using ^1^H-NMR, ^13^C-NMR, and (HR-MS) to confirm the precise structures of the synthesized compounds. Additionally, the molecular docking approach was carried out for most active compounds to explore the binding interactions established by most active compounds, with the active sites of targeted enzymes and obtained results supporting the experimental data.

## 1. Introduction

Alzheimer’s disease (AD), also known as dementia, is a brain disease that affects many people. The condition is characterized by an array of symptoms that include behavioral disruptions, cognitive impairments, a variety of neuropsychiatric disorders, as well as difficulties with daily living activities due to the multifaceted neurodegenerative process. In the United States, Alzheimer’s disease (AD) is considered to be the most common form of dementia. Genetic susceptibility and environmental factors play a major role in this disease’s etiology [1]. Typically, Alzheimer’s disease (AD) affects people over 56 years of age and is generally recognized as a condition associated with aging, resulting in dementia in older individuals. A significant number of individuals aged 65 and older are affected by this condition, which accounts for approximately 10% of the population. As a result, this number increases to more than 30% among those over the age of 80 [2]. In developing nations, Alzheimer’s disease ranks second behind brain accidents, cancer, and cardiovascular diseases in terms of mortality.

AD currently affects 35 million people worldwide, and estimates suggest this number will increase to 107 million by 2050 [2]. The risk factors for cardiovascular disease, such as hypertension and high blood cholesterol levels, are strongly associated with Alzheimer’s disease [3].This process involves the degradation of neurons and the impairment of cholinergic transmission as a result. As a result of Alzheimer’s disease’s multipathogenic nature, a new drug development strategy has been developed. By targeting drugs with multiple actions, such as inhibitors of acetylcholinesterase (AChE) and butyrylcholinesterase (BuChE), this approach is specifically beneficial for treating Alzheimer’s disease [4]. Cognitive processes, particularly memory, depend heavily on acetylcholine, a neurotransmitter. As a result of impaired cholinergic neurotransmission in the brain, Alzheimer’s disease (AD) results in cognitive dysfunction [5,6]. There is a close relationship between the enzyme BuChE and AChE [7]. A growing interest has been generated in its application for treating Alzheimer’s disease because of its potential involvement in the early phases of anticholinesterase therapies [8]. The enzymatic activity of butyrylcholinesterase (BuChE) is commonly enhanced in persons with hyperlipidemia, obesity, and hypertension. The enzymatic activity of BuChE is usually reduced in people treated with beta-blockers or who have recently suffered an acute myocardial infarction [9,10,11]. Neurotransmitter transmission can be negatively affected by inhibiting BuChE because of a decrease in enzymatic activity. There are several symptoms associated with this disorder, including high temperature, blurry vision, nausea, vomiting, dizziness, as well as death [12,13].

The blocking of BuChE, specifically, may lower the likelihood of unfavorable effects and even make it possible to achieve effectiveness while minimizing undesirable consequences [14].

**Figure 1:**
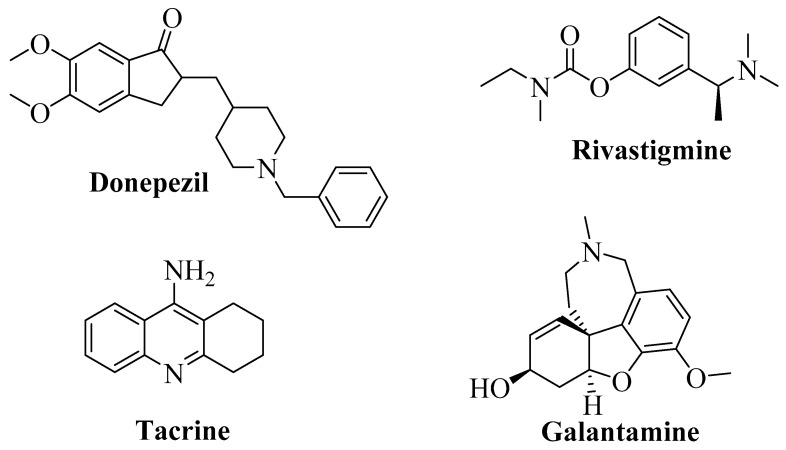
Chemical structure for the reported Alzheimer’s disease drugs.

In order to treat Alzheimer’s disease, pharmaceutical treatments have increasingly focused on the selective inhibition of BuChE. A combination of selective cholinesterase inhibitors and ongoing BuChE inhibitors may enhance clinical results in the future [15], as shown in Figure 1. Indazole, which belongs to the azoles group, contains carbon, nitrogen, and hydrogen. Benzpyrazole or isoindazolone are common names for this molecule. The compound consists of two nitrogen atoms and is classified as a heterocyclic organic compound. As a ten-pi-electron aromatic heterocycle, indazole exhibits similar properties to pyrazole, pyridine, and pyrrole. Numerous biological compounds and medicinal preparations rely on indazole for frameworks. Thus, chemists around the world have developed diverse approaches to synthesize these heterocycles. There are significant biological, agricultural, and industrial implications associated with indazole, an example of a heterocycle [16,17]. Previous studies have provided evidence of the diverse range of activities exhibited by indazole and its derivatives. These activities encompass anti-HIV, anti-inflammatory, anti-platelet, and 5-HT3 receptor properties, as well as anti-tumor and antagonistic effects [18,19]. Indazole binds strongly to the 5-HT1A receptor. Moreover, it has a high affinity for the I2 receptor. The pharmacological properties of this compound make the development of enhanced synthetic methodologies highly imperative [20]. Biologically active pharmaceutical compounds are commonly derived from 1,3,4-thiadiazoles. This demonstrates that pharmacologically active scaffolds can be designed and developed with many intriguing biological applications. Other thanthese activities, five-membered heterocyclic thiadiazole scaffolds have been reported to possess antioxidant properties [21,22], antidepressants [23,24], antileishmanial properties [25], glucuronidase inhibitory properties [26,27], urease inhibitory, and antifungal properties [28,29]. We developed a plan to design and synthesize indazole-containing thiadiazole derivatives as a result of the persistence of our research groups. As acetylcholinesterase inhibitors (AChE) and butyrylcholinesterase (BuChE) inhibitors, these derivatives are intended to be a novel class of compounds.

## 2. Results and Discussion

### 2.1. Chemistry

**Scheme 1:**
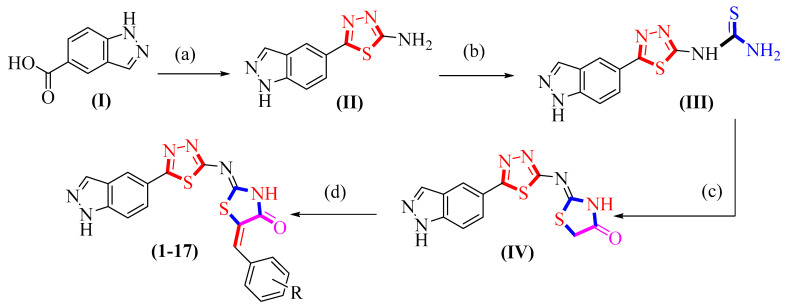
Synthetic procedure and reaction condition for the synthesis of indazole-based thiadiazole-bearing thiazolidinone derivatives (**1**–**17**). (**a**) Thiosemicarbazide, POCl_3_, Reflux 4 h; (**b**) NH_4_NCS, EtOH/Et_3_N, Reflux 4 h; (**c**) Thioglycolic acid, EtOH/CH_3_COOH, Reflux 8 h; (**d**) Benzaldehyde, EtOH/Et_3_N, Reflux 5 h.

Indazole-based thiadiazole (**II**) was synthesized by refluxing 1H-indazole-5-carboxylic acid (**I**) with thiosemicarbazide in the presence of phosphorous oxychloride for four hours. The synthesized thiadiazole (**II**) derivative was reacted with ammonium isothiocyanate in the presence of triethylamine in ethanol and refluxed for four hours and then indazole-based thiourea derivative (**III**) was synthesized. Indazole-based thiadiazole-carrying thiazolidinone derivatives (**IV**) were synthesized by subjecting a specific compound to a reaction with thioglycolic acid in the presence of a small amount of acetic acid in an ethanol solvent for a duration of eight hours. The intermediate (**IV**) was subjected to reflux conditions and reacted with benzaldehyde derivatives with varying substitution patterns in response to the presence of a small amount of triethylamine (Et_3_N) in ethanol for a duration of 5 h. This reaction yielded a series of indazole-based thiadiazole-bearing thiazolidinone moieties (**1**–**17**), as discussed below in Scheme 1. The synthesized compounds were confirmed using HREIMS, ^13^CNMR and ^1^H NMR (see Supplementary Information).

### 2.2. In Vitro Acetylcholinesterase (AChE) Inhibitory Potential

The inhibitory potentials of the synthesized derivatives (**1**–**17**) based on indazole-based thiadiazole were assessed against AChE, as documented in the literature. The compounds exhibited a diverse range of acetylcholinesterase (AChE) inhibition, with values ranging from (0.86 ± 0.33 μM) to (26.73 ± 0.84 μM), in comparison to Donepezil (IC_50_ = 1.26 ± 0.18 μM), which served as the reference inhibitor. On the other hand, among the indazole-based thiadiazole scaffolds (**1**–**17**) screened, namely scaffolds 1, 9, 14, 15, and 16, these scaffolds showed a significant inhibitory effect on the acetylcholinesterase enzyme. The IC_50_ values for these scaffolds were determined to be 1.10 ± 0.37, 0.86 ± 0.30, 0.92 ± 0.10, 1.24 ± 0.36, and 1.20 ± 0.58 μM, respectively. It was determined that the results of the experiment were similar to the IC_50_ value of the ordinary drug Donepezil. As a result of the work carried out on the analogs, it was observed that certain compounds showed inhibitory effects that were moderate to relatively low, which were within the range of the reference medication, within the cohort of compounds under investigation. Despite this, it was observed that the inhibitory potentials of the compounds that were tested were reduced in comparison with the inhibitory potentials of the Donepezil reference drugs, as shown in Table 1.

Taking into account the results of the structure–activity relationship (SAR) investigation, it is evident that the activity of the synthesized compounds (**1**–**17**) against acetylcholinesterase (AChE) is primarily determined by the properties of the phenyl ring attached to the thiadiazole ring, including the type, position, arrangement, and nature of substitutions within the ring. The molecular structure of newly synthesized derivatives of the indazole-based 1,3,4-thiadiazole compounds, together with their respective IC_50_ values, are thoroughly analyzed in order to determine the unique characteristics thathave been impacted by the addition of a trifluoro group to the phenyl ring linked to the 1,3,4-thiadiazole moiety. Accordingly, it appears that the derivatives of 1,3,4-thiadiazoles, including the indazole moiety, that is, those containing a trifluoro group, are more inhibitory against acetylcholinesterase in comparison with analogous derivatives of these compounds. As a result of the presence of the trifluoro group located at the *para*-position of the phenyl ring, compound **9** (IC_50_ = 0.86 ± 0.30 μM) was determined to be the most potent analog within this series. The compound exhibited notable efficacy in comparison to the conventional Donepezil (IC_50_ = 1.26 ± 0.18 μM).

The -CF_3_ introduces electron-withdrawing properties to the phenyl ring attached to thethiadiazole skeleton, as well as its unique electronic effect that enhances lipophilicity. Based on the results of the experiment, it has been determined that it will definitely decrease the electron density and increase the biological potency of the scaffolds as well. The increased efficacy of scaffold **9** (IC_50_ = 0.86 ± 0.30 μM) against acetylcholinesterase might, perhaps, be related to the participation of the trifluoro group in forming hydrogen bonds with the active region of the enzyme.

**Figure 2:**
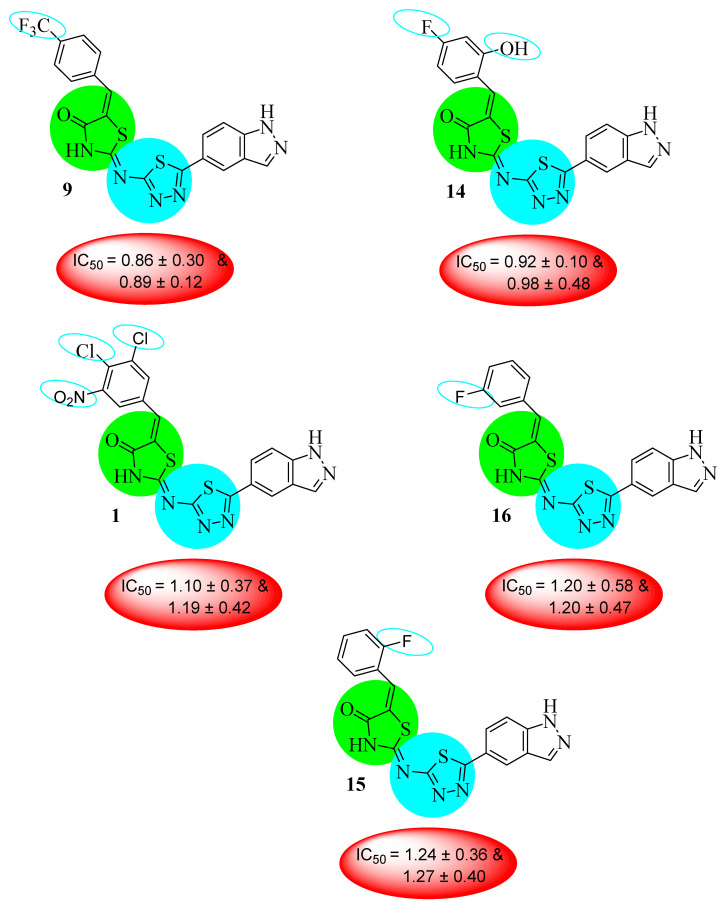
Structure of the potent scaffolds of synthesized indazole-based thiadiazole-bearing thiazolidinone derivatives (**9**, **14**, **1**, **16**, and **15**).

There has been significant evidence that compound **14**, which is characterized by the presence of *ortho*-hydroxy and *para*-fluoro substitution groups in the phenyl ring attached to the thiadiazole moiety, is highly effective against the acetylcholinesterase enzyme. Compound **14** with an IC_50_ value of 0.92 ± 0.10 was identified as the second most powerful compound in the series. As a result of the fluorine atoms being involved in the formation of hydrogen bonds with the enzyme active site, it is known that substitutions with fluorine can lead to the introduction of electron-withdrawing groups to the phenyl rings, thereby altering a compound’s metabolic stability, lipophilicity, and bioactivity. When compared with *ortho* or *meta* positions, the *para*-position is often more advantageous for steric hindrance minimization, so it is a more strategic position. Moreover, the *ortho*-OH substitution is basically polar in nature, which allows it to create stronger hydrogen bonds with the active sites of the enzymes, thereby increasing the pharmacokinetic properties of the scaffolds in terms of drug delivery. One of the downsides of the *ortho*-position is that it leads to steric hindrance as well as changes in molecular geometry, as shown in Figure 2.

Structure **14** exhibits greater efficacy against the acetylcholinesterase enzyme when compared to structures **15** and **16**, as seen by its lower IC_50_ value of 0.92 ± 0.10. In contrast, Structure **15** exhibits an IC_50_ value of 1.24 ± 0.36, while Structure **16** has an IC_50_ value of 1.20 ± 0.58. It is likely that the observed phenomenon is due to the significant interaction between the hydroxyl (-OH) and fluorine (-F) groups on the phenyl moiety attached to the 1,3,4-thiadiazole ring. In comparison with analog **1**, compound **8** displayed relatively diminished activity due to the presence of a dichloro phenyl ring. The second molecule, which has anIC_50_ value of 1.10 ± 0.37, has higher potential due to the inclusion of both di-chloro and -NO_2_ groups, which interact with the active site of the acetylcholinesterase enzyme. At *meta* positions, chlorine atoms can affect steric hindrance to some extent, influencing a molecule’s inhibitory potential. An aromatic ring’s electronic properties are moderately influenced by its *meta* position. It can influence electron density distribution within the ring, influencing overall enzymatic activities. Similarly, the nitro group is a strong electron-withdrawing group. This results in a decrease in electron density on the aromatic ring as it pulls electron density away from the ring. Consequently, the ring becomes less nucleophilic and more electrophilic due to this electron-withdrawal effect. So, the nitro group and -Cl group might increase the inhibitory potential of the synthesized compounds. When compared to compounds **1** and **8**, compound **2**, which contains an *ortho*-chloro-substituted phenyl ring connected to an indazole-thiadiazole ring, exhibited reduced inhibitory potency against acetylcholinesterase. Among the analogs 1,3,4-thiadazoles are linked to an *ortho*-chloro group on the phenyl ring, while analog **8** is linked to an *ortho*-alkyl group. The IC_50_ value of compound **2** was found to be 3.11 ± 0.59, indicating its inhibitory efficacy. Conversely, compound **7**, which contains an electron-withdrawing -NO_2_ in the *para*-position, significantly showed a similar potential against both AChE and BuChE, as shown in Figure 2.

Compound **10** (IC_50_ = 24.68 ± 0.12), compound **11** (IC_50_ = 26.73 ± 0.84), compound **12** (IC_50_= 25.12 ± 0.18), and compound **17** (IC_50_ = 22.30 ± 1.00) demonstrate comparable inhibitory properties due to the presence of a bromo group on the phenyl ring connected to the 1,3,4-thiadiazole moiety in each of these compounds. Compound **17**, characterized by the presence of a nitro group in the *meta*-position on the phenyl ring attached to the 1,3,4-thiadiazole ring, has comparatively superior effectiveness against the acetylcholinesterase enzyme when compared to **10**, **11**, and **12** scaffolds but shows less effectiveness as compared to the standard drugs. The observed augmentation in activity can be attributed to the electron-withdrawing characteristics of the -NO_2_ moiety. Compounds **3**, **4**, **5**, and **6**, which possess a methoxy group and a methyl group at the *ortho*, *meta*, and *para*-positions, respectively, have a diminished inhibitory capacity (IC_50_ values of 8.26 ± 0.40, 8.60 ± 1.05, 10.43 ± 0.98, and 12.73 ± 0.33) in comparison to the reference medication Donepezil. In a similar vein, it was demonstrated that compound **13**, which features a naphthalene ring, exhibited no discernable activity, as shown in Table 1.

### 2.3. In Vitro Butyrylcholinesterase (BuChE) Inhibitory Potential

In this study, thiadiazole derivatives of indazole (**1**–**17**) were evaluated against butyrylcholinesterase (BuChE) according to reported methodologies. The compounds demonstrated a wide spectrum of butyrylcholinesterase (BuChE) inhibition, as seen by their IC_50_ values, which varied from 0.89 ± 0.12 μM to 27.08 ± 0.19 μM. The range of values obtained for the inhibitor under investigation was compared to those of the standard inhibitor, Donepezil, which exhibited an IC_50_ value of 1.35 ± 0.37 μM. Among the seventeen scaffolds that were subjected to testing, it was observed that five specific scaffolds (scaffolds **1**, **9**, **14**, **15**, and **16**) demonstrated noteworthy inhibitory effects on the enzyme butyrylcholinesterase (BuChE). The IC_50_ values for these scaffolds were determined to be 1.19 ± 0.42, 0.89 ± 0.12, 0.98 ± 0.48, 1.27 ± 0.40, and 1.20 ± 0.47 micromolar (μM), respectively. According to the results, the IC_50_ value for the reference drug Donepezil is similar to the observed values. The remaining twelve compounds were somewhat equivalent, and some compounds showed lower inhibitory potential as compared to standard Donepezil. There were, however, some analogs with moderate inhibitory potential. Through a thorough analysis of the molecular structure of newly synthesized derivatives of indazole-based 1,3,4-thiadiazole compounds and their respective IC_50_ values, it was observed that the incorporation of a trifluoro group onto the phenyl group attached to the 1,3,4-thiadiazole ring led to a significant improvement in their intrinsic properties. Thus, indazole-based derivatives, including 1,3,4-thiadiazole, specifically with trifluoro, *para*-fluoro, di-chloro, and nitro substituents, significantly suppress butyrylcholinesterase (BuChE) as compared to analogous compounds. These groups are important because they have good binding interactions with enzyme-active domains. Compound **9**, with an IC_50_ value of 0.89 ± 0.12 μM, was identified as the most efficacious derivative in this series owing to the inclusion of a *para*-positioned tri-fluoro group adjacent to the phenyl ring. The molecule has significant effectiveness similar to the well-established standard Donepezil, as evidenced by its reported IC_50_ value of 1.35 ± 0.37 μM. The enhanced efficacy of alternative scaffolds **1** (IC_50_ = 1.19 ± 0.42 μM), 14 (IC_50_ = 1.10 ± 0.37 μM), 15 (IC_50_ = 1.27 ± 0.40 μM), and 16 (IC_50_ = 1.20 ± 0.47 μM) against butyrylcholinesterase (BuChE) may potentially be attributed to the presence of fluoro, di-chloro, nitro, and hydroxy groups, which have the ability to establish hydrogen bonds with the active site of the targeted enzyme, as shown in Table 1.

### 2.4. Docking Study of the Synthesized Compounds (***1***–***17***)

**Figure 3:**
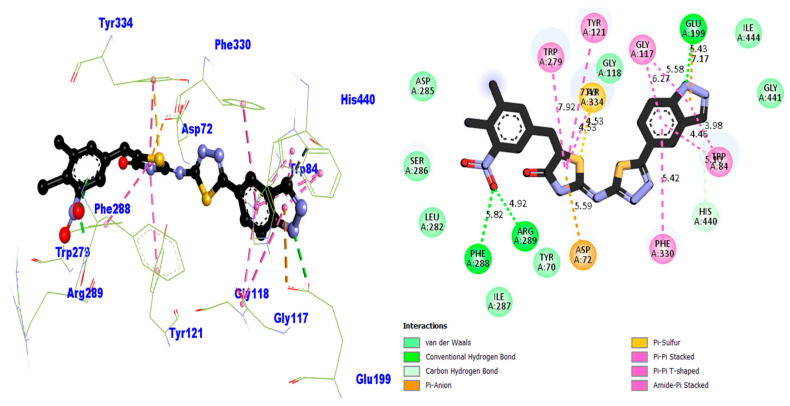
Compound **1**’s closest interactions with AChE inhibitor active site residues.

**Figure 4:**
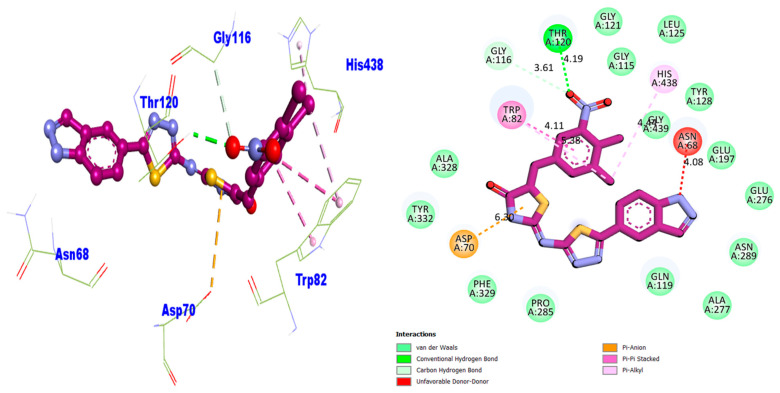
Compound **1**’s closest interactions with BuChE inhibitor active site residues.

**Figure 5:**
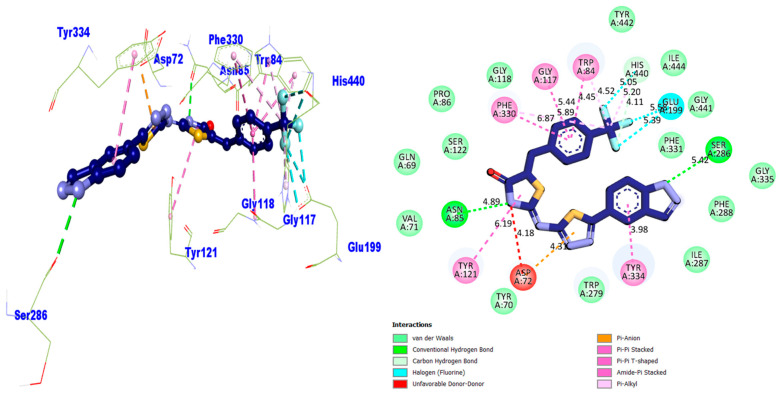
Compound **9**’s closest interactions with AChE inhibitor active site residues.

**Figure 6:**
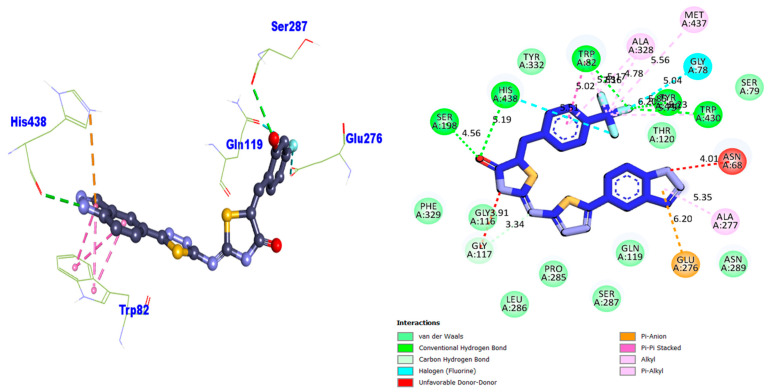
Compound **9**’s closest interactions with BuChE inhibitor active site residues.

**Figure 7:**
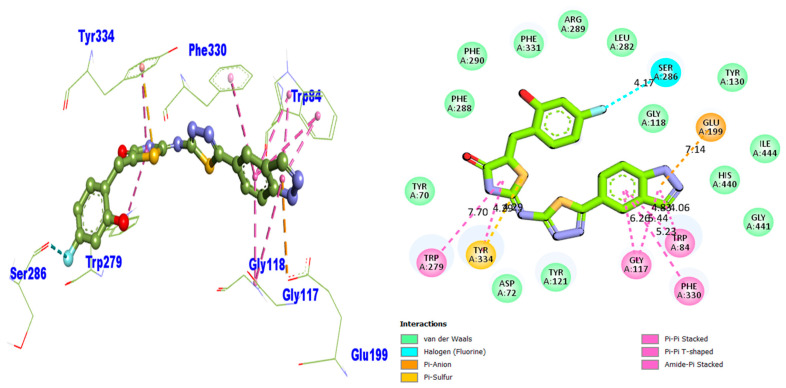
Compound **14**’s closest interactions with AChE inhibitor active site residues.

**Figure 8:**
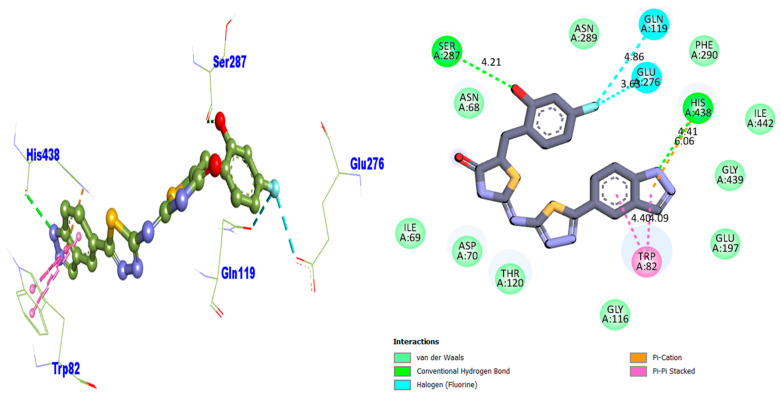
Compound **14**’s closest interactions with BuChE inhibitor active site residues.

The acetylcholinesterase and butyrylcholinesterase inhibitions of the synthesized derivatives of indazole-based thiadiazole-bearing thiazolidinone derivatives (**1**–**17**) are shown in Table 1. The IC_50_ values of indazole-based thiadiazole derivatives indicate that the inhibition of acetylcholinesterase is greatly affected by the specific positions, types, and nature of substituted functional groups on the aromatic ring of the core structure. A molecular docking study was conducted using the docking tools Auto Dock Vina, Discovery Studio Visualizer (DSV), and Pymol, keeping the co-ordinate configuration of X = 18.221339, Y= 7.684984, Z = 49.559016 for AchE and co-ordinate configuration of X = 0.105784, Y = −2.213477, Z = −22.937114 for BuChE to obtain insight into the observed enzymatic inhibition of the synthesized derivatives and to assess the influence of different parameters, such as the type, number, and position of substituted functional groups, on cholinesterase (ChE) inhibition. The objective of this investigation was to determine the specific ways in which the synthesized compounds interacted with the active site residues of AChE and BuChE. The significance of the *ortho*, *meta*, and *para* locations of the substituted functional group on the inhibition of acetylcholinesterase, as quantified by IC_50_ values, is noteworthy. The aforementioned sites have a significant impact on both the number and characteristics of the intermolecular interactions that take place between the substituted functional group and the active amino acids present in acetylcholinesterase. Compounds **1**, **9**, **14**, and **16** have structural differences in the positioning of the substituted trifluoro, fluorine, chlorine, and hydroxy groups at the *ortho*, *para*, and *meta* positions, correspondingly. The experimental results suggest that compound **9** has a higher level of inhibition of acetylcholinesterase in comparison to compounds **1**, **14**, and **16**. The observed result aligns with a greater frequency of interactions taking place between the trifluoro group located at the *para*-position of compound **9**, as opposed to compounds **1** and **14**. Electronegative elements, including fluorine (-F), chlorine (-Cl), and nitrogen (-N) atoms, are known to have strong interactions with various active sites of amino acids, as shown in Figure 3, Figure 4, Figure 5, Figure 6, Figure 7 and Figure 8, with their respective receptor, types of interaction, and their docking score, as shown in Table 2.

## 3. Materials and Method

### 3.1. General Information

A large number of chemical reagents and chemicals were purchased from Sigma-Aldrich and Alfa Aesar for the purposes of this study. On the basis of an Advance Bruker spectrometer, NMR spectra of ^1^H and ^13^C in DMSO-*d6* were recorded at 600 MHz and 150 MHz, respectively. An LCMS-IT-TOF system from Schimadzu Japan (Kyoto, Japan) was used to obtain mass spectra for the analysis of the samples. The melting point of the sample was determined by using the B-chi melting point-560 apparatus. A silica gel GF_254_ coating was applied to aluminum plates in order to monitor the progress of the reaction.

### 3.2. General Procedure for the Synthesis of Indazole-Based Thiadiazole-Bearing Thiazolidinone Scaffold

In the first step, indazole-based thiadiazole (**I**) was synthesized by refluxing 1H-indazole-5-carboxylic acid (1 mmol) with thiosemicarbazide (1mmol) in the presence of phosphorous oxychloride (30 mL) for four hours. The synthesized thiadiazole (**II**) (1 mmol) was reacted further with ammonium isothiocyanate (1 mmol) in the presence of triethylamine (two to three drops) in ethanol and refluxed for four hours, and indazole-based thiourea derivative (**III**) was synthesized, which was further reacted with thioglycolic acid (1 mmol) in the presence of few drops of acetic acid in ethanol for eight hours.Indazole-derived thiadiazole-based thiazolidinone derivative (**IV**) was synthesized. The intermediate (**IV**) (1 mmol) was further reacted under reflux with variously substituted benzaldehyde (1 mmol) in the presence of a few drops of Et_3_N in ethanol for 5 h and with indazole-based thiadiazole-bearing thiazolidinone derivatives (**1**–**17**). The reaction was monitored through TLC. 

### 3.3. Spectral Analysis

#### 3.3.1. (Z)-2-((5-(1H-Indazol-5-yl)-1,3,4-Thiadiazol-2-yl)Imino)-5-((E)-3,4-Dichloro-5-Nitrobenzylidene)Thiazolidin-4-One (**1**)

Yield 69%, off-white solid, M.P 228–229 °C, ^1^H-NMR (600 MHz, DMSO-*d6*): δ 12.27 (s, 1H, N-H), 11.86 (s, 1H, N-H), 8.81 (d, *J*= 7.0 Hz, 1H, H_Ar_), 8.54 (s, 1H, H_Ar_), 8.37 (s, 1H, H_Ar_), 8.22 (d, *J*= 6.9 Hz, 1H, H_Ar_), 7.93 (s, 1H, H_Ar_), 7.84 (s, 1H, H_alkene_), 7.68 (s, 1H, H_Ar_); ^13^C-NMR (150 MHz, DMSO-*d6*): δ 177.6, 174.2, 168.6, 168.3, 162.8, 160.1, 157.3, 152.7, 147.1, 147.0, 142.9, 141.7, 138.4, 136.1, 136.0, 131.3, 130.9, 125.2, 120.4; HREI-MS: *m*/*z* calcld for C_19_H_9_N_7_Cl_2_O_3_S_2_, [M]^+^ 518.4344 Found 518. 4332.

#### 3.3.2. (Z)-2-((5-(1H-Indazol-5-yl)-1,3,4-Thiadiazol-2-yl)Imino)-5-((E)-2-Chlorobenzylidene)Thiazolidin-4-One (**2**)

Yield 73%, pale-yellow solid, M.P 213–214 °C, ^1^H-NMR (600 MHz, DMSO-*d6*): δ 12.29 (s, 1H, N-H), 11.92 (s, 1H, N-H), 8.61 (d, *J* = 7.3 Hz, 1H, H_Ar_), 8.44 (s, 1H, H_Ar_), 8.39 (s, 1H, H_Ar_), 8.31 (d, *J*= 6.4 Hz, 1H, H_Ar_), 8.11 (dd, *J* = 6.7, 1.3 Hz, 1H, H_Ar_), 7.89 (s, 1H, H_alkene_), 7.74 (dd, *J* = 7.0, 1.9 Hz, 1H, H_Ar_), 7.68 (t, *J* = 6.9 Hz, 2H, H_Ar_); ^13^C-NMR (150 MHz, DMSO-*d6*): δ 173.4, 171.6, 168.5, 166.4, 159.4, 156.7, 154.3, 151.2, 150.3, 148.4, 145.3, 142.1, 140.3, 136.4, 132.9, 130.6, 129.7, 126.1, 121.5; HREI-MS: *m*/*z* calcld for C_19_H_11_ClN_6_OS_2_, [M]^+^ 438.6539 Found 438. 6526.

#### 3.3.3. (Z)-2-((5-(1H-Indazol-5-yl)-1,3,4-Thiadiazol-2-yl)Imino)-5-((E)-2,4-Dimethylbenzylidene)Thiazolidin-4-One (**3**)

Yield 57%, off-white solid, M.P 205–206 °C, ^1^H-NMR (600 MHz, DMSO-*d6*): δ 12.37 (s, 1H, N-H), 11.91 (s, 1H, N-H), 8.73 (d, *J* = 7.1 Hz, 1H, H_Ar_), 8.67 (s, 1H, H_Ar_), 8.43 (s, 1H, H_Ar_), 8.39 (d, *J*= 6.3 Hz, 1H, H_Ar_), 8.12 (d, *J* = 6.8 Hz, 1H, H_Ar_), 7.90 (s, 1H, H_alkene_), 7.78 (s, 1H, H_Ar_), 7.69 (d, *J* = 6.4 Hz, 1H, H_Ar_), 2.34 (s, 6H, CH_3_); ^13^C-NMR (150 MHz, DMSO-*d6*): δ 178.3, 176.2, 169.7, 162.4, 159.4, 154.2, 152.6, 150.7, 150.1, 144.5, 141.8, 139.5, 139.0, 134.7, 131.6, 130.4, 129.4, 121.6, 118.2, 32.7, 32.3; HREI-MS: *m*/*z* calcld for C_21_H_16_N_6_OS_2_, [M]^+^ 442.4087 Found 442.4078.

#### 3.3.4. (Z)-2-((5-(1H-Indazol-5-yl)-1,3,4-Thiadiazol-2-yl)Imino)-5-((E)-3,5-Dimethylbenzylidene)Thiazolidin-4-One (**4**)

Yield 64%, off-white solid, M.P 208–209 °C, ^1^H-NMR (600 MHz, DMSO-*d6*): δ 12.16 (s, 1H, N-H), 11.87 (s, 1H, N-H), 8.87 (d, *J* = 7.1 Hz, 1H, H_Ar_), 8.45 (s, 1H, H_Ar_), 8.31 (s, 1H, H_Ar_), 8.23 (d, *J*= 7.0 Hz, 1H, H_Ar_), 7.86 (s, 1H, H_Ar_), 7.81 (s, 1H, H_alkene_), 7.61 (s, 1H, H_Ar_), 7.51 (s, 1H, H_Ar_), 2.47 (s, 6H, CH_3_); ^13^C-NMR (150 MHz, DMSO-*d6*): δ 175.6, 173.3, 169.6, 168.2, 163.8, 159.1, 157.3, 153.8, 148.1, 145.5, 141.9, 140.8, 139.7, 137.4, 136.8, 132.3, 131.9, 127.3, 119.3, 20.5, 20.2; HREI-MS: *m*/*z* calcld for C_21_H_16_N_6_OS_2_, [M]^+^ 432.4134 Found 432. 4125.

#### 3.3.5. (Z)-2-((5-(1H-Indazol-5-yl)-1,3,4-Thiadiazol-2-yl)Imino)-5-((E)-4-Methoxybenzylidene)Thiazolidin-4-One (**5**)

Yield 78%, off-white solid, M.P 211–212 °C, ^1^H-NMR (600 MHz, DMSO-*d6*): δ 12.37 (s, 1H, N-H), 11.99 (s, 1H, N-H), 8.76 (d, *J*= 7.3 Hz, 1H, H_Ar_), 8.64 (d, *J* = 6.3 Hz, 1H, H_Ar_), 8.55 (s, 1H, H_Ar_), 8.49 (s, 1H, H_Ar_), 7.91 (d, *J*= 6.5 Hz, 2H, H_Ar_), 7.62 (s, 1H, H_alkene_), 7.57 (d, *J*= 6.4 Hz, 2H, H_Ar_), 3.51 (s, 3H, -OCH_3_); ^13^C-NMR (150 MHz, DMSO-*d6*): δ 180.4, 176.3, 167.2, 164.4, 163.5, 161.5, 161.4, 158.6, 156.9, 153.4, 152.1, 146.1, 143.6, 141.9, 141.4, 138.4, 134.9, 127.5, 123.6, 55.8; HREI-MS: *m*/*z* calcld for C_20_H_14_N_6_O_2_S_2_, [M]^+^ 434.2760 Found 434.2749.

#### 3.3.6. (Z)-2-((5-(1H-Indazol-5-yl)-1,3,4-Thiadiazol-2-yl)Imino)-5-((E)-3-Methoxybenzylidene)Thiazolidin-4-One (**6**)

Yield 70%, off-white solid, M.P 218–219 °C, ^1^H-NMR (600 MHz, DMSO-*d6*): δ 12.37 (s, 1H, N-H), 11.90 (s, 1H, N-H), 8.78 (d, *J*= 7.0 Hz, 1H, H_Ar_), 8.63 (s, 1H, H_Ar_), 8.51 (s, 1H, H_Ar_), 8.34 (d, *J*= 6.8 Hz, 1H, H_Ar_), 7.81 (dd, *J* = 7.0, 2.2 Hz, 1H, H_Ar_), 7.61 (s, 1H, H_alkene_), 7.63 (t, *J*= 7.0 Hz, 1H, H_Ar_), 7.53 (s, 1H, H_Ar_), 7.49 (dd, *J*= 7.1, 2.3 Hz, 1H, H_Ar_); ^13^C-NMR (150 MHz, DMSO-*d6*): δ 177.8, 175.1, 164.2, 161.8, 157.2, 156.2, 153.1, 152.0, 150.7, 149.4, 148.1, 144.7, 143.8, 139.1, 137.2, 134.0, 127.6, 126.3, 121.8, 54.8; HREI-MS: *m*/*z* calcld for C_20_H_14_N_6_O_2_S_2_, [M]^+^ 434.7276 Found 434.7261.

#### 3.3.7. (Z)-2-((5-(1H-Indazol-5-yl)-1,3,4-Thiadiazol-2-yl)Imino)-5-((E)-4-Nitrobenzylidene)Thiazolidin-4-One (**7**)

Yield 81%, light-yellow solid, M.P 222–223 °C, ^1^H-NMR (600 MHz, DMSO-*d6*): δ 12.33 (s, 1H, N-H), 11.93 (s, 1H, N-H), 8.72 (d, *J* = 7.0 Hz, 1H, H_Ar_), 8.64 (d, *J* = 7.0 Hz, 1H, H_Ar_), 8.57 (s, 1H, H_Ar_), 8.52 (s, 1H, H_Ar_), 7.81 (d, *J* = 6.0 Hz, 2H, H_Ar_), 7.61 (s, 1H, H_alkene_), 7.53 (d, *J* = 6.8 Hz, 2H, H_Ar_); ^13^C-NMR (150 MHz, DMSO-*d6*): δ 178.5, 171.8, 163.8, 162.7, 160.4, 160.2, 153.6, 153.6, 151.9, 150.7, 149.7, 147.7, 144.6, 143.7, 139.6, 136.7, 131.7, 121.8, 119.9; HREI-MS: *m*/*z* calcld for C_19_H_11_N_7_O_3_S_2_, [M]^+^ 449.1756 Found 449.1741.

#### 3.3.8. (Z)-2-((5-(1H-Indazol-5-yl)-1,3,4-Thiadiazol-2-yl)Imino)-5-((E)-3,4-Dichlorobenzylidene)Thiazolidin-4-one (**8**)

Yield 62%, yellow solid, M.P 218–219 °C, ^1^H-NMR (600 MHz, DMSO-*d6*): δ 12.31 (s, 1H, N-H), 11.89 (s, 1H, N-H), 8.71 (d, *J*= 7.0 Hz, 1H, H_Ar_), 8.59 (s, 1H, H_Ar_), 8.43 (s, 1H, H_Ar_), 8.39 (d, *J* = 6.7 Hz, 1H, H_Ar_), 7.87 (d, *J*= 6.8 Hz, 1H, H_Ar_), 7.81 (s, 1H, H_alkene_), 7.70 (s, 1H, H_Ar_), 7.63 (d, *J*= 6.8 Hz, 1H, H_Ar_); ^13^C-NMR (150 MHz, DMSO-*d6*): δ 175.7, 173.8, 168.1, 161.7, 159.4, 156.2, 154.1, 152.7, 151.8, 146.2, 144.1, 140.1, 140.0, 137.6, 134.6, 133.2, 131.1, 127.4, 122.8; HREI-MS: *m*/*z* calcld for C_19_H_10_Cl_2_N_6_OS_2_, [M]^+^ 473.4312 Found 473.4303.

#### 3.3.9. (Z)-2-((5-(1H-Indazol-5-yl)-1,3,4-Thiadiazol-2-yl)Imino)-5-((E)-4-(Trifluoromethyl)Benzylidene)Thiazolidin-4-One (**9**)

Yield 62%, white solid, M.P 226–227 °C, ^1^H-NMR (600 MHz, DMSO-*d6*): δ 12.42 (s, 1H, N-H), 11.83 (s, 1H, N-H), 8.75 (d, *J* = 6.5 Hz, 1H, H_Ar_), 8.56 (s, 1H, H_Ar_), 8.49 (s, 1H, H_Ar_), 8.24 (d, *J* = 7.0 Hz, 1H, H_Ar_), 7.92 (d, *J* = 7.0 Hz, 2H, H_Ar_), 7.81 (s, 1H, H_alkene_), 7.62 (d, *J* = 6.5 Hz, 2H, H_Ar_); ^13^C-NMR (150 MHz, DMSO-*d6*): δ 171.3, 169.7, 166.1, 164.0, 161.4, 156.1, 155.9, 151.8, 150.9, 150.2, 146.2, 141.7, 147.6, 141.6, 134.5, 132.0, 130.7, 125.6, 122.4, 119.1; HREI-MS: *m*/*z* calcld for C_20_H_11_F_3_N_6_OS_2_, [M]^+^ 472.2731 Found 472.2718.

#### 3.3.10. (Z)-2-((5-(1H-Indazol-5-yl)-1,3,4-Thiadiazol-2-yl)Imino)-5-((E)-4-Bromobenzylidene)Thiazolidin-4-One (**10**)

Yield 78%, off-white solid, M.P 231–232 °C, ^1^H-NMR (600 MHz, DMSO-*d6*): δ 12.28 (s, 1H, N-H), 11.72 (s, 1H, N-H), 8.63 (d, *J* = 7.0 Hz, 1H, H_Ar_), 8.45 (s, 1H, H_Ar_), 8.39 (s, 1H, H_Ar_), 8.12 (d, *J* = 7.2 Hz, 1H, H_Ar_), 7.89 (d, *J* = 7.3 Hz, 2H, H_Ar_), 7.74 (s, 1H, H_alkene_), 7.58 (d, *J* = 6.8 Hz, 2H, H_Ar_); ^13^C-NMR (150 MHz, DMSO-*d6*): δ 177.9, 174.9, 169.4, 166.8, 160.5, 157.3, 156.6, 154.8, 150.3, 147.6, 143.1, 142.8, 140.6, 140.2, 136.2, 133.7, 133.4, 128.5, 117.6; HREI-MS: *m*/*z* calcld for C_19_H_11_BrN_6_OS_2_, [M]^+^ 483.2341 Found 483.2330.

#### 3.3.11. (Z)-2-((5-(1H-Indazol-5-yl)-1,3,4-Thiadiazol-2-yl)Imino)-5-((E)-3-Bromobenzylidene)Thiazolidin-4-One (**11**)

Yield 60%, off-white solid, M.P 226–227 °C, ^1^H-NMR (600 MHz, DMSO-*d6*): δ 12.32 (s, 1H, N-H), 11.87 (s, 1H, N-H), 8.79 (d, *J* = 6.3 Hz, 1H, H_Ar_), 8.63 (s, 1H, H_Ar_), 8.52 (s, 1H, H_Ar_), 8.29 (d, *J* = 7.1 Hz, 1H, H_Ar_), 7.86 (dd, *J* = 6.8, 2.1 Hz, 1H, H_Ar_), 7.74 (s, 1H, H_alkene_), 7.62 (t, *J* = 7.3 Hz, 1H, H_Ar_), 7.51 (s, 1H, H_Ar_), 7.43 (dd, *J* = 7.1, 2.3 Hz, 1H, H_Ar_); ^13^C-NMR (150 MHz, DMSO-*d6*): δ 179.8, 177.2, 161.2, 160.4, 156.7, 153.1, 150.1, 150.0, 149.6, 141.4, 139.3, 134.1, 130.4, 130.1, 127.5, 124.8, 121.6, 118.5, 112.1; HREI-MS: *m*/*z* calcld for C_19_H_11_BrN_6_OS_2_, [M]^+^ 483.9229 Found 483.9217.

#### 3.3.12. (Z)-2-((5-(1H-Indazol-5-yl)-1,3,4-Thiadiazol-2-yl)Imino)-5-((E)-3-Bromo-5-Methylbenzylidene)Thiazolidin-4-One (**12**)

Yield 65%, off-white solid, M.P 229–230 °C, ^1^H-NMR (600 MHz, DMSO-*d6*): δ 12.23 (s, 1H, N-H), 11.92 (s, 1H, N-H), 8.58 (d, *J* = 6.2 Hz, 1H, H_Ar_), 8.43 (s, 1H, H_Ar_), 8.39 (s, 1H, H_Ar_), 8.26 (d, *J* = 7.2 Hz, 1H, H_Ar_), 7.93 (s, 1H, H_Ar_), 7.84 (s, 1H, H_alkene_), 7.64 (s, 1H, H_Ar_), 7.52 (s, 1H, H_Ar_), 2.34 (s, 3H, CH_3_); ^13^C-NMR (150 MHz, DMSO-*d6*): δ 174.1, 170.0, 167.2, 164.8, 159.1, 153.2, 150.6, 150.4, 149.9, 144.7, 141.1, 140.0, 139.1, 137.0, 133.9, 131.3, 130.5, 128.4, 118.6, 30.5; HREI-MS: *m*/*z* calcld for C_20_H_13_BrN_6_OS_2_, [M]^+^ 497.4089 Found 497. 4081.

#### 3.3.13. (2Z,5E)-2-((5-(1H-Indazol-5-yl)-1,3,4-Thiadiazol-2-yl)Imino)-5-(Naphthalen-1-Ylmethylene)Thiazolidin-4-One (**13**)

Yield 63%, white solid, M.P 221–222 °C, ^1^H-NMR (600 MHz, DMSO-*d6*): δ 12.27 (s, 1H, N-H), 12.03 (s, 1H, N-H), 8.41 (d, *J* = 7.2 Hz, 1H, H_Ar_), 8.54 (s, 1H, H_Ar_), 8.51 (s, 1H, H_Ar_), 8.43 (dd, *J* = 6.5 Hz, 1H, H_Ar_), 8.23 (dd, *J*= 6.9, 1.3 Hz, 1H, H_Ar_), 8.18 (d, *J*= 6.7 Hz, 1H, H_Ar_), 8.13 (dd, *J*= 6.7, 1.4 Hz, 1H, H_Ar_), 8.01 (t, *J* = 6.7 Hz, 1H, H_Ar_), 7.89 (s, 1H, Halkene), 7.64 (dd, *J* = 6.5, 1.8 Hz, 1H, H_Ar_), 7.59 (t, *J* = 7.1 Hz, 1H, H_Ar_), 7.59 (t, *J*= 7.1 Hz, 1H, HAr); ^13^C-NMR (150 MHz, DMSO-*d6*): δ 177.6, 176.3, 164.8, 163.9, 159.1, 158.4, 155.1, 153.8, 152.3, 149.4, 147.8, 144.7, 140.4, 138.5, 136.8, 134.4, 132.9, 127.5, 125.8, 123.8, 123.2, 119.2, 115.7; HREI-MS: *m*/*z* calcld for C_23_H_14_N_6_OS_2_, [M]^+^ 454.2150 Found 454. 2143.

#### 3.3.14. (Z)-2-((5-(1H-Indazol-5-yl)-1,3,4-Thiadiazol-2-yl)Imino)-5-((E)-4-Fluoro-2-Hydroxybenzylidene)Thiazolidin-4-One (**14**)

Yield 69%, light-yellow solid, M.P 218–219 °C, ^1^H-NMR (600 MHz, DMSO-*d6*): δ 12.13 (s, 1H, N-H), 11.31 (s, 1H, N-H), 10.34 (s, 1H, OH), 8.51 (d, *J* = 6.8 Hz, 1H, H_Ar_), 8.39 (s, 1H, H_Ar_), 8.37 (s, 1H, H_Ar_), 8.19 (d, *J* = 7.0 Hz, 1H, H_Ar_), 7.89 (d, *J*= 7.2 Hz, 1H, H_Ar_), 7.81 (s, 1H, H_alkene_), 7.60 (s, 1H, H_Ar_), 7.51 (d, *J* = 6.9 Hz, 1H, H_Ar_); ^13^C-NMR (150 MHz, DMSO-*d6*): δ 172.1, 169.8, 164.7, 163.2, 157.2, 154.1, 153.6, 151.4, 150.5, 148.2, 146.1, 144.1, 140.1, 140.0, 139.9, 136.3, 133.5, 122.9, 120.1; HREI-MS: *m*/*z* calcld for C_19_H_11_FN_6_O_2_S_2_, [M]^+^ 438.4345 Found 528.4332.

#### 3.3.15. (Z)-2-((5-(1H-Indazol-5-yl)-1,3,4-Thiadiazol-2-yl)Imino)-5-((E)-2-Fluorobenzylidene)Thiazolidin-4-One (**15**)

Yield 65%, off-white solid, M.P 216–218 °C, ^1^H-NMR (600 MHz, DMSO-*d6*): δ 12.21 (s, 1H, N-H), 11.96 (s, 1H, N-H), 8.63 (d, *J*= 7.1 Hz, 1H, H_Ar_), 8.48 (s, 1H, H_Ar_), 8.44 (s, 1H, H_Ar_), 8.36 (d, *J* = 6.9 Hz, 1H, H_Ar_), 8.02 (dd, *J* = 6.8, 1.2 Hz, 1H, H_Ar_), 7.91 (s, 1H, H_alkene_), 7.83 (dd, *J*= 7.3, 2.0 Hz, 1H, H_Ar_), 7.64 (t, *J*= 6.5 Hz, 2H, H_Ar_); ^13^C-NMR (150 MHz, DMSO-*d6*): δ 175.4, 173.1, 166.5, 164.8, 159.2, 158.7, 159.3, 153.1, 152.3, 150.7, 147.0, 146.1, 143.7, 138.9, 135.8, 131.4, 131.1, 121.4, 120.3; HREI-MS: *m*/*z* calcld for C_19_H_11_FN_6_OS_2_, [M]^+^ 422.4531 Found 422. 4524.

#### 3.3.16. (Z)-2-((5-(1H-Indazol-5-yl)-1,3,4-Thiadiazol-2-yl)Imino)-5-((E)-3-Fluorobenzylidene)Thiazolidin-4-One (**16**)

Yield 83%, off-white solid, M.P 221–222 °C, ^1^H-NMR (600 MHz, DMSO-*d6*): δ 12.19 (s, 1H, N-H), 11.93 (s, 1H, N-H), 8.71 (d, *J*= 7.0 Hz, 1H, H_Ar_), 8.53 (s, 1H, H_Ar_), 8.50 (s, 1H, H_Ar_), 8.39 (d, *J* = 6.7 Hz, 1H, H_Ar_), 7.92 (dd, *J* = 6.9, 1.3 Hz, 1H, H_Ar_), 7.81 (s, 1H, H_alkene_), 7.79 (t, *J* = 7.1 Hz, 1H, H_Ar_), 7.67 (s, 1H, H_Ar_), 7.49 (dd, *J*= 7.0, 2.1 Hz, 1H, H_Ar_); ^13^C-NMR (150 MHz, DMSO-*d6*): δ 178.1, 176.7, 165.2, 163.4, 158.7, 157.1, 154.1, 150.4, 150.3, 145.7, 141.0, 140.7, 140.4, 139.1, 139.0, 135.4, 131.5, 123.4, 121.8; HREI-MS: *m*/*z* calcld for C_19_H_11_FN_6_OS_2_, [M]^+^ 422.6123 Found 422.6111.

#### 3.3.17. (Z)-2-((5-(1H-Indazol-5-yl)-1,3,4-Thiadiazol-2-yl)Imino)-5-((E)-4-Bromo-3-Nitrobenzylidene)Thiazolidin-4-One (**17**)

Yield 74%, yellow solid, M.P 234–235 °C, ^1^H-NMR (600 MHz, DMSO-*d6*): δ 12.25 (s, 1H, N-H), 11.95 (s, 1H, N-H), 8.65 (d, *J* = 6.8 Hz, 1H, H_Ar_), 8.34 (s, 1H, H_Ar_), 8.32 (s, 1H, H_Ar_), 8.09 (d, *J* = 6.9 Hz, 1H, H_Ar_), 7.99 (d, *J* = 6.7 Hz, 1H, H_Ar_), 7.83 (s, 1H, H_alkene_), 7.63 (s, 1H, H_Ar_), 7.51 (d, *J* = 6.0 Hz, 1H, H_Ar_); ^13^C-NMR (150 MHz, DMSO-*d6*): δ 179.2, 175.1, 163.4, 162.9, 159.2, 158.9, 155.2, 151.1, 150.2, 148.7, 143.2, 142.1, 141.2, 141.0, 139.1, 132.2, 130.5, 128.7, 122.4; HREI-MS: *m*/*z* calcld for C_19_H_10_BrN_7_O_3_S_2_, [M]^+^ 528.4107 Found 528.4092.

### 3.4. Inhibition Assay of Anticholinesterase/Butyrylcholinesterase Assay

A spectrophotometric analysis was conducted by Ellman et al. to determine the inhibition of ChEs (AChE and BuChE). The substrates ATCh and BTCh were employed for the purpose of inhibiting the enzymes AChE and BuChE (human sigma 9000-81-1 and 9001-08-5, respectively). An incubation of 15 min was performed at room temperature with 140 L of sodium phosphate buffer (pH 8.0), 20 L of AChE/BuChE solution, and 20 L of the test sample. AChE/BuChE solutions containing DTNB were introduced into the 10 L solution to initiate the reaction. An enzymatic inhibition by AChE and BuChE was not detected during the 15min hydrolysis of DTNB with thiocholine, while ATCh or BTCh acted as catalysts. A percentage of inhibition was calculated using the ratio of enzyme activity in the presence of the test sample (E), minus enzyme activity without the test sample (S), expressed as E–S/E100. Each substance’s inhibition of substrate hydrolysis by ChE was quantified using its IC_50_ value (g/mL) or M. Compounds with the same IC_50_ values are calculated using the same standardized plot. Calculating the IC_50_ value involves equating Y to 50 and then using the x-axis to determine the concentration at which 50% of the inhibitory effect is achieved [30].

### 3.5. Docking Studies

We investigated the binding mechanisms among the indole-based thiadiazole-bearing thiazolidinone derivatives (**1**–**17**) and the active residues of acetylcholinesterase and butyrylcholinesterase using Autodock. The initial docked geometry of acetylcholinesterase and butyrylcholinesterase can be found on the RCSB data bank website (PDB code 4EY7). Co-crystallized structures of receptor–ligand complexes allow for the identification of the active site of acetylcholinesterase and butyrylcholinesterase. A successful replication of the re-docking process for Donepezil into the active site has been achieved, resulting in a root mean square deviation value of 0.63. This was achieved using the Merck molecular force field 94 at level 44. The PDB files that were created from the MMFF94 have then been used to optimize the molecule geometry of the derivatives of the compounds. To determine the binding locations of the original ligand and its synthesized derivatives, a Lamarckian genetic algorithm was used with a total of 500 runs [31,32,33,34].

## 4. Conclusions

In this study, we established a facile protocol for the synthesis of indazole-derived thiadiazole-based thiazolidinone hybrid derivatives (**1**–**17**) by cyclization of indazolethiadiazole-based thiourea substrate with thioglycolic acid, followed by a reaction with benzimidazole. The reaction features, such as high efficiency, mild reaction conditions, simple operation, and short reaction time, make it an attractive alternative for the synthesis of indazole-based 1,3,4-thiadiazole. In addition, all the newly synthesized derivatives were characterized spectroscopically through ^1^HNMR, ^13^CNMR, and HRMS. The synthesized motifs were evaluated in vitro for their acetylcholinesterase (AChE) and butyrylcholinesterase (BuChE) inhibitory activity. All the newly synthesized analogs showed good to moderate inhibitory potentials, ranging from 0.86 ± 0.30 μM to 26.73 ± 0.84 μM and 0.89 ± 0.12 to 27.08 ± 0.12 μM against acetylcholinesterase (AChE) and butyrylcholinesterase (BuChE), respectively. Among the series, compounds **9** (IC_50_ = 0.86 ± 0.30 ΜmandIC_50_ = 0.89 ± 0.12 μM), **14** (IC_50_ = 0.92 ± 0.10 μM and IC_50_ = 0.98 ± 0.48 μM), **1** (IC_50_ = 1.10 ± 0.37 μMandIC_50_ = 1.19 ± 0.42 μM), and **16** (IC_50_ = 1.20 ± 0.58 μM and IC_50_ = 1.20 ± 0.12 μM) having trifluoro, -F, -OH, di-Cl, and -NO_2_ substitution at the *N*-phenyl ring of the indazole-based thiadiazole ring were found to be highly potent inhibitors against acetylcholinesterase (AChE) and butyrylcholinesterase (BuChE) enzymes, respectively. The binding interactions of most active analogs with the active site of enzymes were recognized with the help of molecular docking studies.

## Figures and Tables

**Table 1:** Representation of various substitutions on indazole-based thiadiazole-bearing thiazolidinone skeleton along with their respective AChE and BuChE potential (**1**–**17**).

S. No.	R	AChE IC_50_ ± SEM (µM)	BuChE IC_50_ ± SEM (µM)
**1**	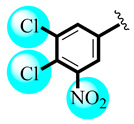	1.10 ± 0.37	1.19 ± 0.42
**2**	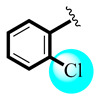	3.11 ± 0.59	3.36 ± 0.28
**3**	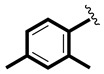	8.26 ± 0.40	8.80 ± 0.13
**4**	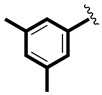	8.60 ± 1.05	8.84 ± 0.98
**5**	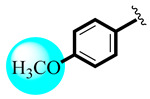	10.43 ± 0.98	10.62 ± 0.74
**6**	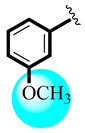	12.73 ± 0.33	12.98 ± 0.15
**7**	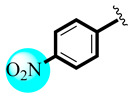	1.40 ± 0.31	1.57 ± 0.28
**8**	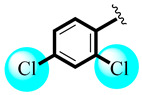	1.22 ± 0.64	1.29 ± 0.41
**9**	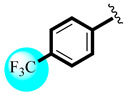	0.86 ± 0.30	0.89 ± 0.12
**10**	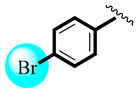	24.68 ± 0.12	25.03 ± 0.44
**11**	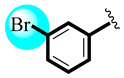	26.73 ± 0.84	27.08 ± 0.19
**12**	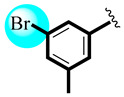	25.12 ± 0.18	26.20 ± 0.67
**13**	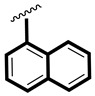	N.A.	N.A.
**14**	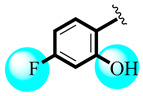	0.92 ± 0.10	0.98 ± 0.48
**15**	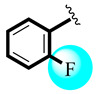	1.24 ± 0.36	1.27 ± 0.40
**16**	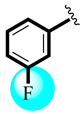	1.20 ± 0.58	1.20 ± 0.47
**17**	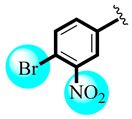	22.30 ± 1.00	20.46 ± 0.86
Standard drug Donepezil		1.26 ± 0.18 µM	1.35 ± 0.37 µM

**Table 2:** In silico inhibitory potential of AChE and BuChE of indazole-based thiadiazole-bearing thiazolidinone scaffolds (**1**, **9** and **14**).

Active Analog	Distance (A°)	Type of Interaction	Receptor	Docking Score
Compound **1** AChE	5.82	Conventional H–B	PHE-A-288	−11.86
	4.92	Conventional H–B	ARG-A-289	
	5.59	Pi–Anion	ASP-A-72	
	5.42	Pi–Pi Stacked	PHE-A-330	
	5.10	Carbon H–B	HIS-A-440	
	4.46	Pi–Pi Stacked	TRP-A-84	
	3.98	Pi–Pi T-shaped	TRP-A-84	
	7.17	Pi–Anion	GLU-A-199	
	5.43	Conventional H–B	GLU-A-199	
	5.58	Pi–Pi T-shaped	GLY-A-117	
	6.27	Pi–Pi Stacked	GLY-A-117	
	4.53	Pi–Sulfur	TYR-A-334	
	4.53	Pi–Pi T-shaped	TYR-A-334	
	7.49	Pi–Pi T-shaped	TYR-A-121	
	7.92	Pi–Pi T-shaped	TRP-A-279	
Compound **1** BuChE	6.30	Pi–Anion	ASP-A-70	−7.56
	4.08	Unfavorable D–D	ASN-A-68	
	4.44	Pi–Alkyl	HIS-A-438	
	4.19	Conventional H–B	THR-A-120	
	3.61	Carbon H–B	GLY-A-116	
	4.11	Pi–Pi Stacked	TRP-A-82	
	5.38	Pi–Alkyl	TRP-A-82	
Compound **9** AChE	4.89	Conventional H–B	ASN-A-85	−10.34
	6.19	Amide–Pi Stacked	TYR-A-121	
	4.18	Unfavorable D–D	ASP-A-72	
	4.31	Pi–Anion	ASP-A-72	
	3.98	Pi–Pi Stacked	TYR-A-334	
	5.42	Conventional H–B	SER-A-286	
	5.39	H–F	GLU-A-199	
	5.58	H–F	GLU-A-199	
	4.11	Carbon H–B	HIS-A-440	
	5.20	Pi–Alkyl	HIS-A-440	
	5.05	H–F	HIS-A-440	
	4.52	Pi–Alkyl	TRP-A-84	
	4.45	Pi–Pi Stacked	TRP-A-84	
	5.44	Pi–Pi Stacked	GLY-A-117	
	5.89	Pi–Alkyl	PHE-A-330	
	6.87	Pi–Pi Stacked	PHE-A-330	
Compound **9** BuChE	3.91	Unfavorable D–D	GLY-A-117	−12.88
	3.34	Carbon H–B	GLY-A-117	
	6.20	Pi–Anion	GLU-A-276	
	5.35	Pi–Alkyl	ALA-A-277	
	4.01	Unfavorable D–D	ASN-A-68	
	5.75	Alkyl	TRP-A-430	
	5.23	Conventional H–B	TRP-A-430	
	6.20 5.86	Alkyl Conventional H–B	TYR-A-440 TYR-A-440	
	5.04	H–F	GLY-A-78	
	5.56	Alkyl	MET-A-437	
	4.78	Alkyl	ALA-A-328	
	5.17	Pi–Alkyl	ALA-A-328	
	5.36	Conventional H–B	TRP-A-82	
	5.73	Alkyl	TRP-A-82	
	5.02	Pi–Pi Stacked	TRP-A-82	
	5.51	H–F	HIS-A-438	
	5.19	Conventional H–B	HIS-A-438	
	4.56	Conventional H–B	SER-A-198	
Compound **14** AChE	7.70	Pi–Pi Stacked	TRP-A-279	−6.99
	4.39	Pi–Pi Stacked	TYR-A-334	
	4.29	Pi–Sulfur	TYR-A-334	
	6.26	Pi–Pi Stacked	GLY-A-117	
	5.44	Pi–Pi Stacked	GLY-A-117	
	5.23	Pi–Pi Stacked	PHE-A-330	
	4.83	Pi–Pi Stacked	TRP-A-84	
	4.06	Pi–Pi Stacked	TRP-A-84	
	7.14	Pi–Anion	GLU-A-199	
	4.17	H–F	SER-A-286	
Compound **14** BuChE	4.40	Pi–Pi Stacked	TRP-A-82	−6.12
	4.09	Pi–Pi Stacked	TRP-A-82	
	6.06	Pi–Cation	HIS-A-438	
	4.41	Conventional H–B	HIS-A-438	
	3.63	H–F	GLU-A-276	
	4.86	H–F	GLN-A-119	
	4.21	Conventional H–B	SER-A-287	

## Data Availability

Data is contained within the article and Supplementary Material.

## Supplementary Materials

The following supporting information can be downloaded at: https://www.mdpi.com/article/10.3390/ph16121667/s1, Figure S1: ^13^C NMR for the compound **3** (Z)-2-((5-(1H-indazol-5-yl)-1,3,4-thiadiazol-2-yl)imino)-5-((E)-2,4-dimethylbenzylidene)thiazolidin-4-one. Figure S2: ^1^H NMR for the compound **5** (Z)-2-((5-(1H-indazol-5-yl)-1,3,4-thiadiazol-2-yl)imino)-5-((E)-4-methoxybenzylidene)thiazolidin-4-one. Figure S3: ^13^C NMR for the compound **12** (Z)-2-((5-(1H-indazol-5-yl)-1,3,4-thiadiazol-2-yl)imino)-5-((E)-3-bromo-5-methylbenzylidene)thiazolidin-4-one. Figure S4: ^1^H NMR for the compound **13** (2Z,5E)-2-((5-(1H-indazol-5-yl)-1,3,4-thiadiazol-2-yl)imino)-5-(naphthalen-1-ylmethylene)thiazolidin-4-one. Figure S5: ^13^C NMR for the compound **13** (2Z,5E)-2-((5-(1H-indazol-5-yl)-1,3,4-thiadiazol-2-yl)imino)-5-(naphthalen-1-ylmethylene)thiazolidin-4-one.
