# Supplementary material for: Identification of Indazole-Based Thiadiazole-Bearing Thiazolidinone Hybrid Derivatives: Theoretical and Computational Approaches to Develop Promising Anti-Alzheimer’s Candidates
Source: Pharmaceuticals (Basel). 2023 Nov 30;16(12):1667. doi: 10.3390/ph16121667 (PMC10747300; doi:10.3390/ph16121667)
Supplement: Supplementary file 1 [file pharmaceuticals-16-01667-s001.zip › pharmaceuticals-2717503-SI.pdf]

pharmaceuticals

MDPI

# Identification of Indazole-Based Thiadiazole-Bearing Thiazolidinone Hybrid Derivatives: Theoretical and Computational Approaches to Develop Promising Anti-Alzheimer's Candidates

**Yousaf Khan[1], Shoaib Khan[2], Rafaqat Hussain*[3], Wajid Rehman[3], Aneela Maalik*[1], Urooba Gulshan[1], Mohamed W. Attwa[4], Hany W. Darwish[4], Hazem A. Ghabbour[5] and Nawab Ali[6]**

[1]*Department of Chemistry, COMSATS University Islamabad campus-45550, Islamabad, Pakistan.*
[2]*Department of Chemistry, AbbottabadUniversity of Science and Technology (AUST) Abbottabad, 22500, Pakistan.*
[3]*Department of Chemistry, Hazara University, Mansehra-21120, Pakistan.*
[4]*Department of Pharmaceutical Chemistry, College of Pharmacy, King Saud University, Riyadh 11451, Kingdom of Saudi Arabia*
[5]*School of Health and Biomedical Sciences, RMIT University, Melbourne 3083, Australia*
[6]*Shangai Key Laboratory of Functional Material Chemistry, School of Chemistry and Molecular Engineering, East China University of Science and Technology, Meilong Road 130, Shangai 200237, PR China*
*Correspondence: rafaqathussain0347@gmail.com (R.H) and aneela.maalik@comsats.edu.pk (A.M)

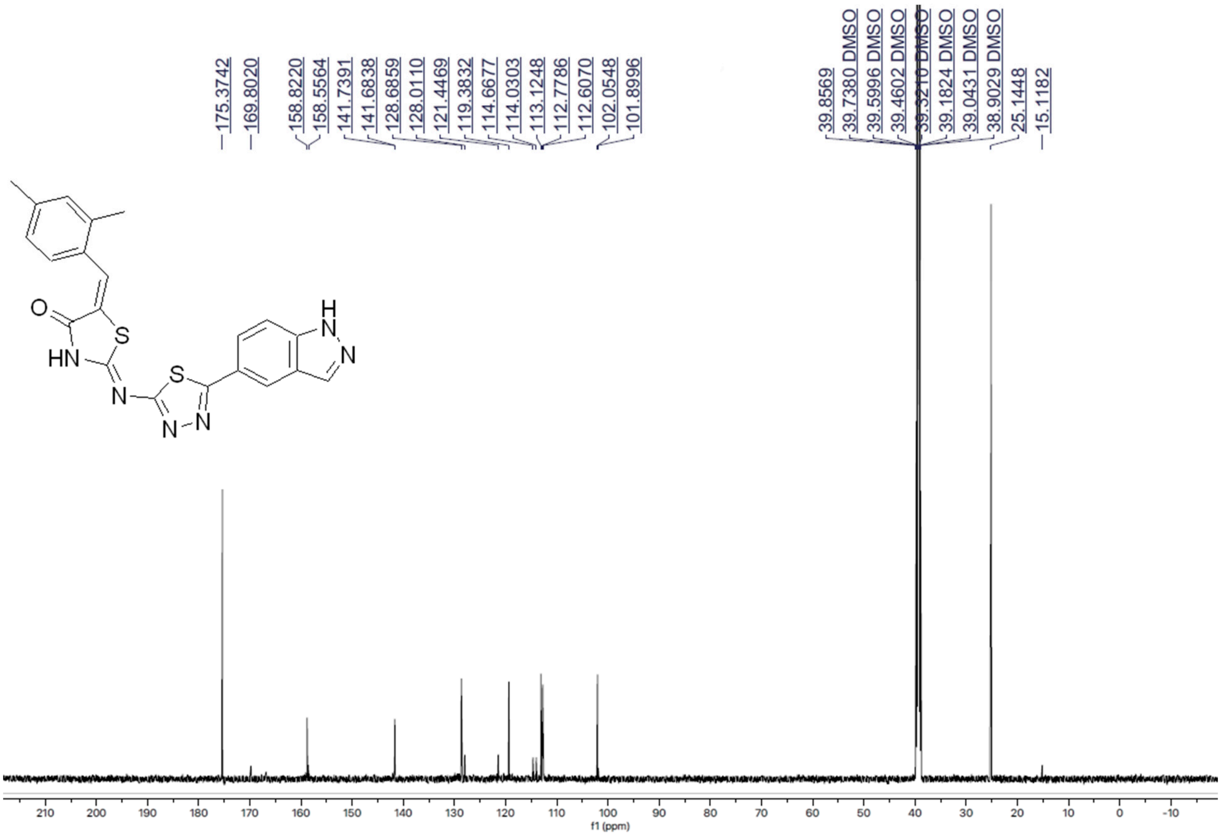


**Figure S1.**$^{13}$C NMR for the compound **3** *(Z)-2-((5-(1H-indazol-5-yl)-1,3,4-thiadiazol-2-yl)imino)-5-((E)-2,4-dimethylbenzylidene)thiazolidin-4-one*

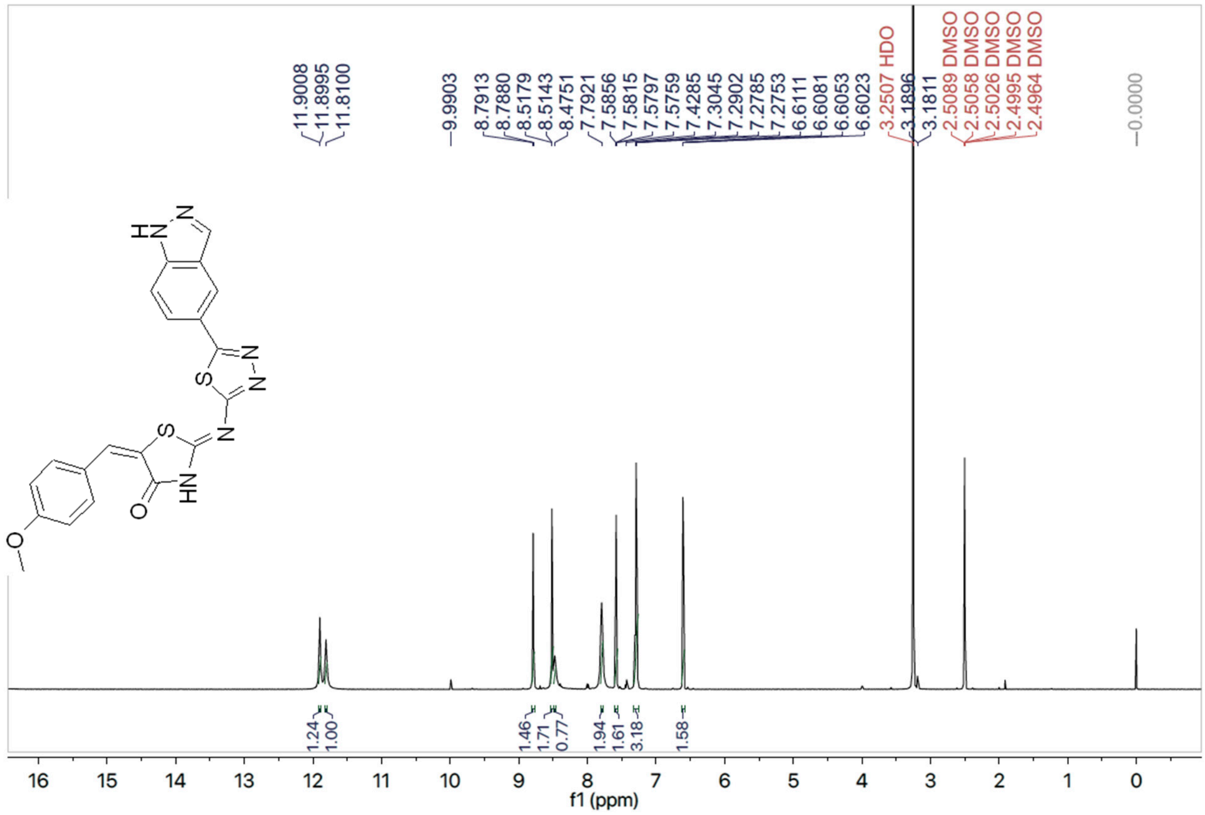


**Figure S2.** $^{1}$H NMR for the compound **5** *(Z)-2-((5-(1H-indazol-5-yl)-1,3,4-thiadiazol-2-yl)imino)-5-((E)-4-methoxybenzylidene)thiazolidin-4-one*

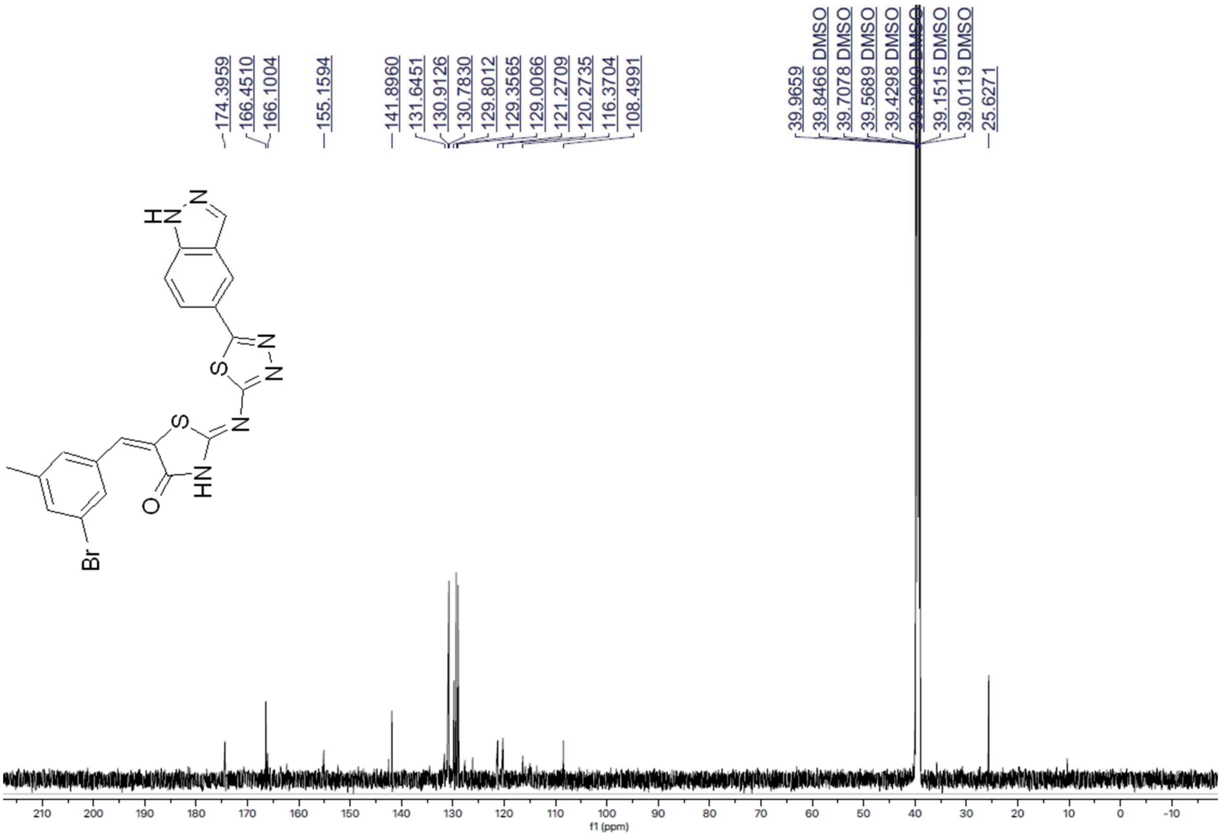


**Figure S3.**$^{13}$C NMR for the compound **12** (Z)-2-((5-(1H-indazol-5-yl)-1,3,4-thiadiazol-2-yl)imino)-5-((E)-3-bromo-5-methylbenzylidene)thiazolidin-4-one

pharmaceuticals

MDPI

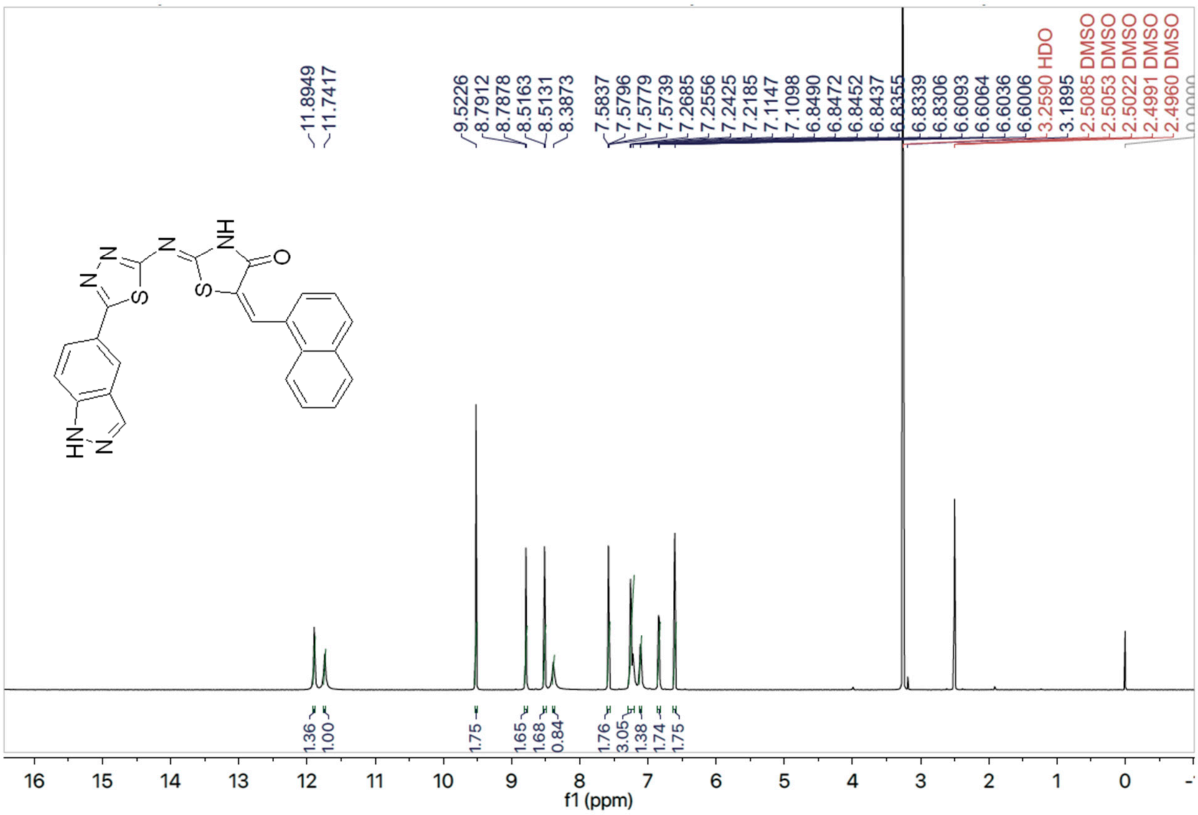


**Figure S4.** $^1$H NMR for the compound **13** *(2Z,5E)-2-((5-(1H-indazol-5-yl)-1,3,4-thiadiazol-2-yl)imino)-5-(naphthalen-1-ylmethylene)thiazolidin-4-one*

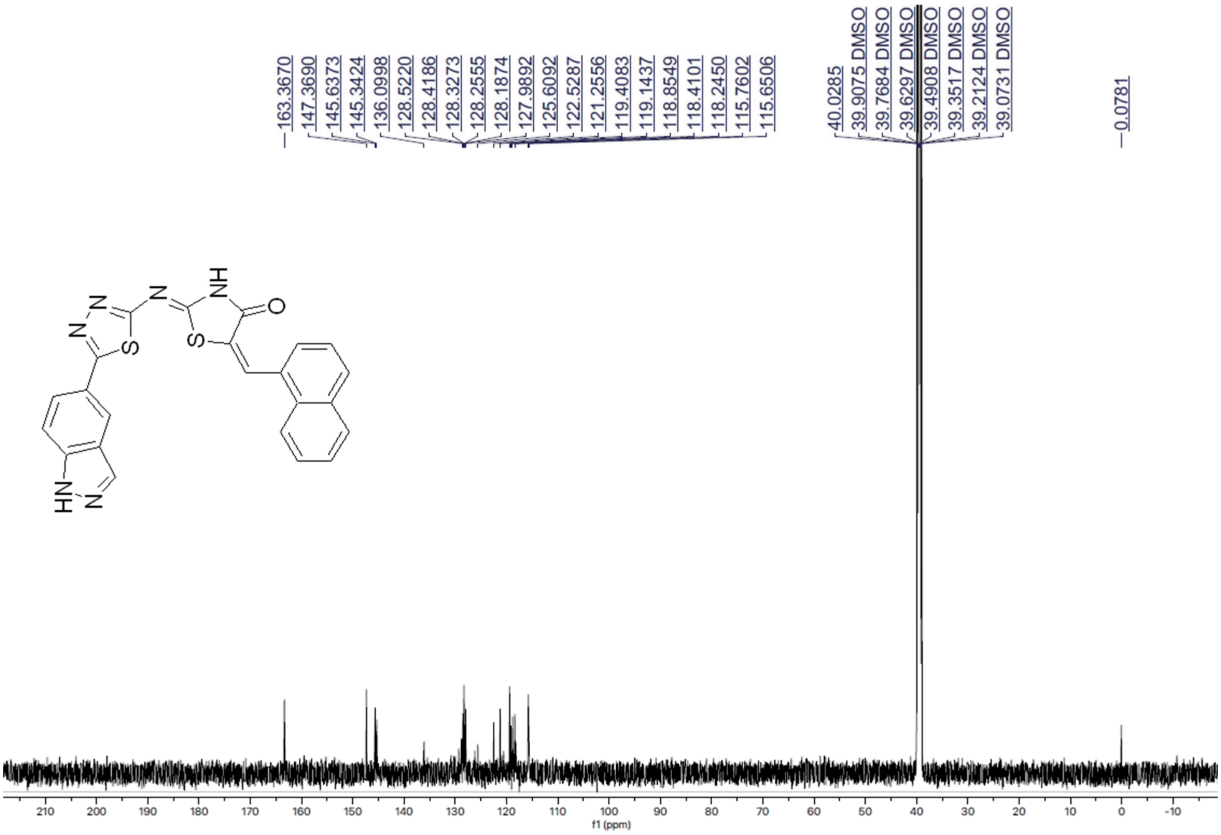


**Figure S5.** $^{13}$C NMR for the compound **13** *(2Z,5E)-2-((5-(1H-indazol-5-yl)-1,3,4-thiadiazol-2-yl)imino)-5-(naphthalen-1-ylmethylene)thiazolidin-4-one*
